# Adherence to a healthy lifestyle behavior composite score and cardiometabolic risk factors in Spanish children from the CORALS cohort

**DOI:** 10.1007/s00431-023-05389-z

**Published:** 2024-01-23

**Authors:** Tany E. Garcidueñas-Fimbres, Carlos Gómez-Martínez, Maria Pascual-Compte, Jose Manuel Jurado-Castro, Rosaura Leis, Luis A. Moreno, Santiago Navas-Carretero, Pilar Codoñer-Franch, Ana Moreira Echeverria, Belén Pastor-Villaescusa, Alicia López-Rubio, Sara Moroño García, Pilar De Miguel-Etayo, J. Alfredo Martínez, Inmaculada Velasco Aguayo, Rocío Vázquez-Cobela, Joaquín Escribano, María Luisa Miguel-Berges, María José De La Torre-Aguilar, Mercedes Gil-Campos, Jordi Salas-Salvadó, Nancy Babio

**Affiliations:** 1 Departament de Bioquímica i Biotecnologia. Unitat de Nutrició Humana, Universitat Rovira i Virgili, Reus, Spain; 2 Institut d’Investigació Sanitària Pere Virgili (IISPV), Reus, Spain; 3 Centro de Investigación Biomédica en Red de Fisiopatología de la Obesidad y Nutrición (CIBEROBN), Instituto de Salud Carlos III (ISCIII), Madrid, Spain; 4 Metabolism and Investigation Unit, Reina Sofia University Hospital, Maimónides Institute of Biomedicine Research of Córdoba (IMIBIC), University of Córdoba, 14004 Córdoba, Spain; 5 Unit of Pediatric Gastroenterology, Hepatology and Nutrition, Pediatric Service, Hospital Clínico Universitario de Santiago, 15706 Santiago de Compostela, Spain; 6 Pediatric Nutrition Research Group, Unit of Investigation in Nutrition, Growth and Human Development of Galicia-USC, Health Research Institute of Santiago de Compostela (IDIS), 15706 Santiago de Compostela, Spain; 7 Growth, Exercise, Nutrition and Development (GENUD) Research Group, University of Zaragoza, Zaragoza, Spain; 8 Instituto de Investigación Sanitaria de Aragón (IIS Aragón), Instituto Agroalimentario de Aragón (IA2), Zaragoza, Spain; 9 Center for Nutrition Research, University of Navarra, 31008 Pamplona, Spain; 10 Fac Pharm & Nutr, Dept Nutr Food Sci & Physiol, University of Navarra, 31008 Pamplona, Spain; 11 IdisNA, Navarra Institute for Health Research, Pamplona, Spain; 12 Department of Pediatrics, Obstetrics and Gynecology, Dr. Peset University Hospital, University of Valencia, Valencia, Spain; 13 Fundació Hospital Sant Joan de Deu Martorell, Barcelona, Spain; 14 EDP Salut Sant Joan Baix Camp-ABS Riudoms, Riudoms, Spain; 15 Paediatrics, Nutrition, and, Development Research Unit, Hospital Universitari Sant Joan de Reus, Universitat Rovira i Virgili, Reus, Spain

**Keywords:** Lifestyle behaviors, Eating speed, Childhood obesity, Cardiometabolic risk, CORALS

## Abstract

To assess the associations between the adherence to a composite score comprised of 6 healthy lifestyle behaviors and its individual components with several cardiometabolic risk factors in Spanish preschool children. Cross-sectional analyses were conducted in 938 participants included in the CORALS cohort aged 3–6 years. Six recognized healthy lifestyle behaviors (breastfeeding, sleep duration, physical activity, screentime, adherence to the Mediterranean diet, and eating speed) were assessed in a composite score. Multiple linear and logistic regression models were fitted to assess the associations with cardiometabolic risk factors (weight status, waist circumference, fat mass index, blood pressure, fasting plasma glucose, and lipid profile). In the adjusted multiple linear and logistic regression models, compared with the reference category of adherence to the healthy lifestyle behavior composite score, those participants in the category of the highest adherence showed significant decreased prevalence risk of overweight or obesity [OR (95% CI), 0.4 (0.2, 0.6)] as well as significant lower waist circumference, fat mass index (FMI), systolic blood pressure and fasting plasma glucose concentration [*β* (95% CI), − 1.4 cm (− 2.5, − 0.4); − 0.3 kg/m^2^ (− 0.5, − 0.1); and − 3.0 mmHg (− 5.2, − 0.9); − 1.9 mg/dL (− 3.5, − 0.4), respectively]. Slow eating speed was individually associated with most of the cardiometabolic risk factors.

*   Conclusions*: Higher adherence to the healthy lifestyle behavior composite score was associated with lower waist circumference, FMI, other cardiometabolic risk factors, and risk of overweight or obesity in Spanish preschool children. Further studies are required to confirm these associations.

**Table:** 

**What is Known:** *• Lifestyle is a well-recognized etiologic factor of obesity and its comorbidities.* *• Certain healthy behaviors such as adhering to a healthy diet, increasing physical activity, and decreasing screentime are strategies for prevention and treatment of childhood obesity.*	
**What is New:** *• Higher adherence to the healthy lifestyle behavior composite score to 6 healthy behaviors (breastfeeding, sleep duration, physical activity, screentime, eating speed, and adherence to the Mediterranean diet) was associated with decreased adiposity, including prevalence risk of overweight or obesity, and cardiometabolic risk in preschool children.* *• Slow eating and greater adherence to the Mediterranean diet were mainly associated to lower fasting plasma and serum triglycerides concentration, respectively.*

**Supplementary Information:**

The online version contains supplementary material available at 10.1007/s00431-023-05389-z.

## Introduction

Overweight and obesity is a global Public Health concern. The highest prevalence in Europe is observed in Mediterranean and Eastern European countries [1]. The ALADINO 2019 study [2] reported, in Spanish children aged 6 to 9 years, a prevalence of overweight and obesity of 23.3% and 17.3%, respectively. Furthermore, it is estimated that in 2030, around 9 million children of 5 to 9 years old will have obesity in Europe [3].

Obesity is the consequence of a complex bio-socioecological framework in which intrapersonal factors, lifestyle behaviors, among others, interact [4, 5], where socioeconomic environment is considered an important determinant of the disease at the community level that could explain differences obesity prevalence between regions and countries [6, 7]. Childhood overweight and obesity has been associated with several long-term cardiometabolic disorders in adulthood [5]. In this sense, consistently adiposity status from childhood to adulthood has been associated with higher risk of diabetes, hypertension, and lipid profile disorders [5].

Several modifiable lifestyle behaviors have been associated with obesity or cardiometabolic risk factors such as sedentary behaviors, lower levels of moderate-to-vigorous physical activity, unhealthy dietary patterns (including energy-dense and micronutrient-poor foods), reduced sleep duration, and certain early life factors, among others [4]. Benefits from breastfeeding have been reported [8], so that its duration was inversely associated with cardiometabolic risk [9] and weight gain in childhood. Adherence to the Mediterranean diet (MedDiet) and eating speed have been related to adiposity and cardiometabolic risk; however, evidence in children is very limited [10, 11]. In recent decades, evidence has emerged on adherence to “a priori” lifestyle scores including diet, sleep, physical activity, or screentime, which have been related to lower adiposity or certain cardiometabolic risk factors in children [12–15]. However, none of these composite scores have included breastfeeding [9], eating speed [16, 17], or adherence to the MedDiet [18, 19]. Hence, the aim of the present study was to assess cross-sectionally the relationships between adherence to a composite score comprised of 6 lifestyle behaviors (breastfeeding, sleep duration, physical activity, screentime, adherence to the MedDiet, and eating speed) and its individual components with several cardiometabolic risk factors in children aged 3 to 6 years.

## Methods

### Study design and participants

Cross-sectional analyses were conducted in the Childhood Obesity Risk Assessment Longitudinal Study (CORALS). CORALS recruitment began in May 2019, and due to the COVID-19 pandemic, we were unable to reach the calculated sample size (*n* = 2214). Consequently, recruitment concluded in June 2022 with 1509 participants. Despite this, considering a significance level of 0.01, a small effect size (Cohen’s *d* = 0.2), and a statistical power of 0.8, the suggested sample size was 584 participants, which we significantly exceeded for the purposes of this study. A detailed description of the CORALS can be found elsewhere [11].

For the present study, the inclusion criteria were (a) having available data on the duration of main meals (breakfast, lunch and dinner), physical activity, breastfeeding, sleep duration, the 18-item questionnaire of adherence to the MedDiet and screentime, and biochemical parameters and blood pressure and (b) having reported plausible energy intake on food frequency questionnaires (FFQs). Participants with current diagnosis of chronic diseases, including type 2 diabetes mellitus, hypertension, and familiar hypercholesterolemia were excluded.

**Fig. 1:**
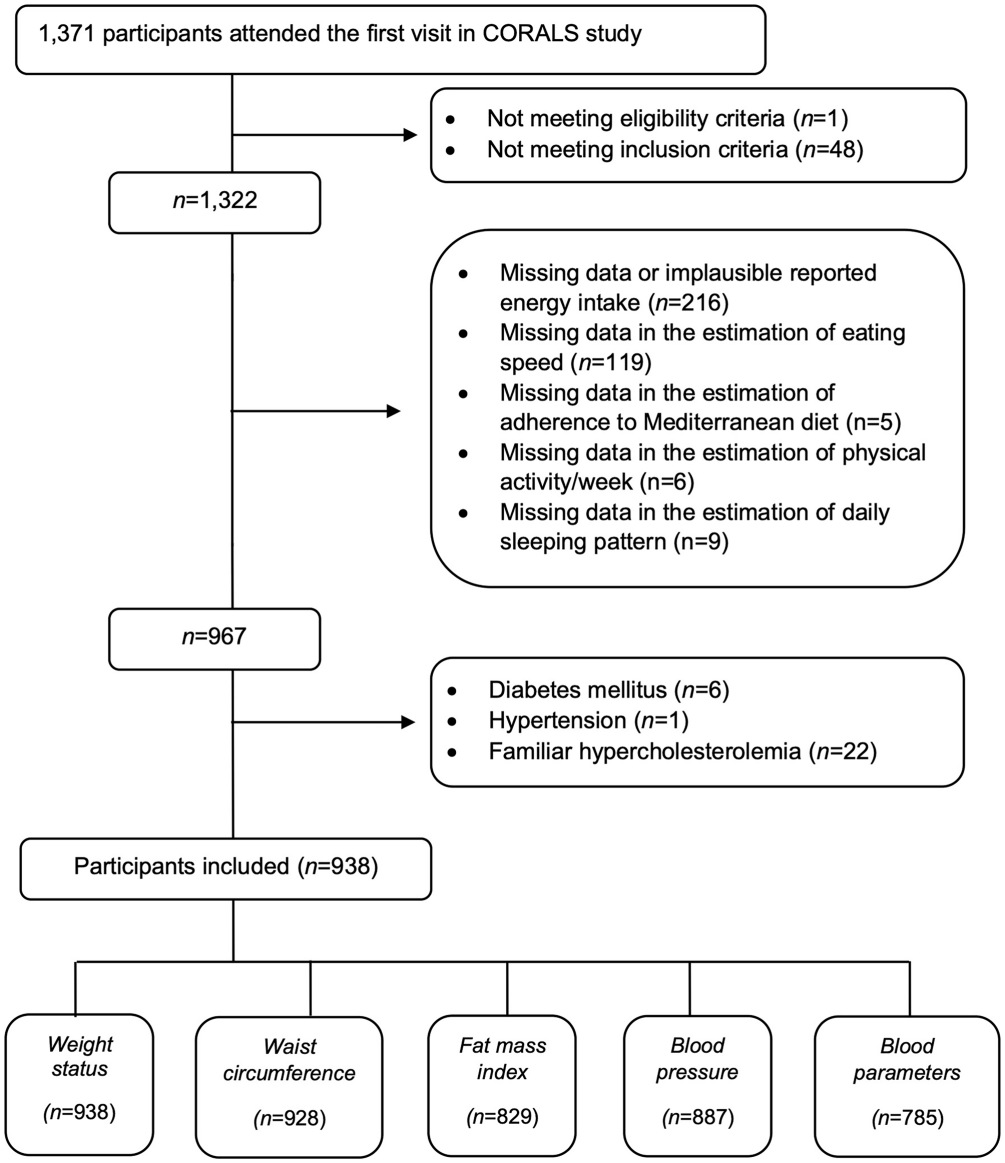
Flow diagram for CORALS participants

A total of 1371 participants attended the CORALS baseline visit, of which 49 participants were excluded from the analyses for not meeting eligibility and inclusion criteria as well as 216 participants for having missing data or implausible reported energy intake. In addition, 139 participants were no longer included due to missing data on ≥ 1 variables required to estimate eating speed, adherence to the MedDiet, and total duration of physical activity or daily sleep duration. Finally, 29 participants with current diagnosis of chronic diseases were excluded. The final analyses included 938 children of which 49% were girls (Fig. 1). Questionnaires were filled out by mothers who were main caregivers in the 88% of participants.

### Assessments

Parents or caregivers completed self-administered questionnaires assessing participant data: sociodemographic, dietary characteristics, lifestyle behaviors, early life, and maternal factors, among others. Details on the assessment of confounders and other variables are provided in Supplementary Material, Appendix 1.

#### Exposure variables

##### Breastfeeding

The duration of total breastfeeding (months) was assessed, and participants were categorized according to whether they were exclusively breastfed during the first 6 months of life (yes/no) [20].

##### Sleep duration

The sleep duration was evaluated through the question: “how long does your child sleep at night during weekdays and on weekend days or holidays?” and “how long does your child nap during weekdays and on weekend days or holidays?” Daily sleep duration (hours/day) was calculated [11] and categorized for age in “inadequate or could be adequate” (< 10 h/day or > 13 h/day for children aged 3–5 years and < 9 h/day or > 11 h/day for children aged 6 years) or “adequate” (10–13 h/day for children aged 3–5 years and 9–11 h/day for children aged 6 years) [21].

##### Sports and physical activities

The total time (hours) of sports and physical activities per week was estimated [11], and two categories were created [< 120 min/week (unhealthy behavior) or ≥ 120 min/week (healthy behavior)] [22].

##### Screentime

Screentime for weekdays and weekend days was assessed by two questions: (a) “how long does your child watch television?” and (b) “how long does your child play in the computer/cell phone/game console?” Possible answers were “none,” “0.5–1 h/day,” “1–2 h/day,” “2–3 h/day,” “3–4 h/day,” or “more than 4 h/day.” Total screentime was derived in a quantitative variable, and < 2 h/day was used as compliance with the recommendation [23].

##### Adherence to the MedDiet

An ad hoc 18-item questionnaire adapted to children was used to evaluate adherence to the MedDiet [11]. The total score (0–18 points) was categorized by tertiles, in which the 3rd tertile represented the highest adherence to the MedDiet.

##### Eating speed

Eating speed was estimated by summing the duration of breakfast, lunch, and dinner [11]. Then, it was categorized by tertiles in fast, moderate, and slow eating categories. The slow eating category corresponded to the 3rd tertile, which was considered the healthiest lifestyle behavior.

##### Healthy lifestyle behavior composite score

Participants were categorized for each lifestyle behavior according to compliance (1 point) or not (0 points) with the following indicators: breastfeeding (< 6 months = 0 points; ≥ 6 months = 1 point), sleep duration for age (inadequate/could be adequate = 0 points; adequate = 1 points), sports and physical activities (< 2 h/week = 0 points; ≥ 2 h/week = 1 point), and screentime (< 2 h/day = 1 point; ≥ 2 h/day = 0 points). Eating speed (T1 = 53.8 min/day ± 9; T2 = 76.3 ± 5 min/day; and T3 = 108.3 ± 18 min/day) and adherence to the MedDiet (T1 = 8 ± 1 points; T2 = 11 ± 1 points; and T3 = 14 ± 1 points) were assessed by tertiles (T1 = 0 points, T2 = 0.5 points, and T3 = 1 point). Afterwards, the total score was calculated for each participant by summing the points obtained in each of the lifestyle behaviors (0–6 points). Hereafter, tertiles of combined adherence to these lifestyle behaviors were created (T1 = lowest, T2 = moderate, and T3 = highest).

#### Outcome variables

##### Adiposity

Weight and height were measured by trained registered dietitians. A precision scale (TANITA MS780SMA) was used to measure body weight (kg) and body fat mass (kg). The height and waist circumference (cm) were also evaluated [11], by a portable stadiometer (SECA 213) and a measuring tape (SECA 201), respectively. Weight status was estimated according to body mass index (BMI) and categorized in underweight/normal weight or overweight/obesity according to the cutoff points defined by Cole and Lobstein [24]. The fat mass index (FMI) was calculated as body fat mass (kg)/height (square meters) [25].

##### Cardiometabolic risk factors

Systolic and diastolic blood pressure, fasting plasma glucose, total cholesterol, high-density lipoprotein cholesterol (HDL-c), low-density lipoprotein cholesterol (LDL-c), nonhigh-density lipoprotein cholesterol (non-HDL-c), and triglycerides were assessed.

Blood pressure (mmHg) was measured in the nondominant arm. Total systolic (SBP) and diastolic blood (DBP) pressure values were estimated by the mean of all available data in both arms, if applicable.

Fasting plasma glucose and lipid profile (total cholesterol, HDL-c, LDL-c, and triglycerides) were measured from blood samples collected in fasting conditions. The non-HDL-c (mg/dL) was calculated [26].

## Statistical analyses

CORALS database updated to December 2021 was used. Multiple linear regression models [*β*; 95% confidence interval (CI)] were fitted to assess associations between tertiles of adherence to the healthy lifestyle behavior composite score (exposure), and outcome variables (waist circumference, FMI, SBP and DBP, and lipid profile), except for weight status, for which multiple logistic regression models [odds ratio (OR), 95% confidence interval] were fitted. Associations between each lifestyle behavior and each outcome were also assessed by logistic or linear regression models to assess their individual associations. Models were adjusted by the number of recruited participants in each center, sex, age, mother’s educational level, maternal overweight/obesity status, and birth weight category. Each individual model was further adjusted by the other lifestyle behaviors, except for the one of interest. For all models, the reference category was the 1st tertile or noncompliance with recommendations, according to the case. Analyses were performed in Stata 14 software program (StataCorp), and *p* values < 0.05 were considered statistically significant. Additional details of the statistical analyses are provided in Supplementary Appendix 2.

## Results

Participants had a mean age ± SD of 4.8 ± 1 year-old and showed a 14% and 8% prevalence of overweight and obesity, respectively. The total study population showed a mean of 10.4 ± 1 h/day of sleep duration, 185 ± 115 min/week of physical activity, 1.8 ± 1 h/day of screentime, 11 ± 3 points on the 18-item MedDiet adherence questionnaire (T1 = 8 ± 1 points; T2 = 11 ± 1 points; and T3 = 14 ± 1 points), and 78.1 ± 25 min/day of eating speed in the 3 main meals (T1 = 53.8 min/day ± 9; T2 = 76.3 ± 5 min/day; and T3 = 108.3 ± 18 min/day). The general characteristics of the participants according to the categories of adherence to composite score are shown in Table 1. Mothers of those participants allocated to the 3rd tertile of adherence were more likely to have a higher educational level and lower prevalence of overweight or obesity.

**Table 1:** General characteristics of the studied participants across categories of adherence to the composite score comprised of 6 healthy lifestyle behaviors

	**Tertiles of adherence to the healthy lifestyle behavior composite score**			
	**T1** (< 3 points) *n* = 392	**T2** (3–4 points) *n* = 296	**T3** (> 4 points) *n* = 250	***p* value**
Age, years	4.8 ± 1.0	4.8 ± 1.1	4.9 ± 1.1	0.682
Girls, % (*n*)	49.0 (192)	50.7 (150)	48.4 (121)	0.852
**Early life factors**				
Birth weight, kg	3.3 ± 0.6	3.2 ± 0.6	3.3 ± 0.5	0.677
Birth weight				0.525
Low birth weight, % *(*n)	7.1 (28)	8.5 (25)	5.2 (13)	
Normal birth weight, % (*n*)	86.0 (337)	84.8 (251)	89.6 (224)	
High birth weight, % (*n*)	6.9 (27)	6.8 (20)	5.2 (13)	
Mother weight gain during pregnancy, kg	12.5 ± 4.6	12.5 ± 4.5	12.5 ± 4.6	0.974
**Maternal factors**				
Age, years	40.4 ± 5.5	41.2 ± 5.2	41.3 ± 7.5	0.138
BMI, kg/m^2^	25.7 ± 5.3^ab^	24.6 ± 5.0^a^	23.9 ± 4.2^b^	** < 0.001**
Weight status				** < 0.001**
Underweight or normal weight, % (*n*)	53.1 (208)	65.5 (194)	69.6 (174)	
Overweight or obesity, % (*n*)	46.9 (184)	34.5 (102)	30.4 (76)	
Educational level				** < 0.001**
Primary or lower, % (*n*)	15.1 (59)	9.1 (27)	2.0 (5)	
Secondary, % (*n*)	46.9 (184)	35.8 (106)	34.4 (86)	
Academic—graduated or no reported data, % (*n*)	38.0 (149)	55.1 (163)	63.6 (159)	
Socio-professional category, % (*n*)				0.362
Homemaker/student/retired/unemployed	30.9 (121)	27.4 (81)	26.0 (65)	
Employee	69.1 (271)	72.6 (215)	74.0 (185)	

Data are expressed as mean ± SD or median [IQR] for continuous variables and percentages (numbers) for categorical variables. *p* values were calculated by the chi-square or ANOVA test for categorical and continuous variables, respectively. Values in bold indicate *p* values < 0.05, which were considered significant. Bonferroni’s test for multiple comparisons was used for the results of maternal BMI. Significant differences (*p* value < 0.05) between categories of adherence to the healthy lifestyle behaviors composite score are expressed as follows: *a* = T1 vs. T2; *b* = T1 vs. T3, and *c* = T2 vs. T3

Table 2 shows the characteristics related to lifestyle, adiposity and cardiometabolic risk in participants across the categories of adherence to the composite score. Those children in the top category of adherence shows a lower prevalence of overweight or obesity, FMI, SBP, and DBP (all *p* < 0.05).

**Table 2:** Lifestyle and cardiometabolic risk factors in the studied population across categories of adherence to the composite score comprised of 6 healthy lifestyle behaviors

	**Tertiles of adherence to the healthy lifestyle behavior composite score**			
	**T1** (< 3 points) *n* = 392	**T2** (3–4 points) *n* = 296	**T3** (> 4 points) *n* = 250	***p* value**
Exclusive breastfeeding, % (n)	15.8 (62)	31.4 (93)	71.2 (178)	** < 0.001**
Total sleep duration, hours/day	10.2 ± 1.0^ab^	10.5 ± 0.7^a^	10.6 ± 0.7^b^	** < 0.001**
Sleeping pattern for age				** < 0.001**
Inadequate, % (*n*)	36.2 (142)	11.5 (34)	5.2 (13)	
Adequate, % (*n*)	63.8 (250)	88.5 (262)	94.8 (237)	
Sports and physical activities, minutes/week	154.6 ± 114.1^ab^	195.8 ± 117.7^ac^	220.2 ± 100.4^bc^	** < 0.001**
Healthy behavior (≥ 120 min/week), % (*n*)	53.1 (208)	77.4 (229)	92.4 (231)	** < 0.001**
Screentime, hours/day	2.4 ± 1.1^ab^	1.6 ± 0.9^ac^	1.3 ± 0.6^bc^	** < 0.001**
Healthy behavior (< 2 h/day), % (*n*)	37.8 (148)	75.0 (222)	94.8 (237)	** < 0.001**
Adherence to Mediterranean diet, 0–18 points	9.6 ± 2.5^ab^	10.8 ± 2.6^ac^	12.3 ± 2.4^bc^	** < 0.001**
Eating speed, minutes/day	69.7 ± 22.1^ab^	78.6 ± 23.2^ac^	90.6 ± 26.2^bc^	** < 0.001**
**Adiposity**				
BMI, kg/m^2^	16.7 ± 2.3^b^	16.3 ± 2.0	15.9 ± 1.8^b^	** < 0.001**
Weight status				** < 0.001**
Underweight or normal weight, % (*n*)	70.2 (275)	80.7 (239)	88.4 (221)	
Overweight or obesity, % (*n*)	29.9 (117)	19.3 (57)	11.6 (29)	
Waist circumference, cm	52.6 ± 7.7	52.2 ± 6.3	51.4 ± 5.8	0.103
Fat mass index, kg/m^2^	4.1 ± 1.4^ab^	3.8 ± 1.2^a^	3.6 ± 1.2^b^	** < 0.001**
**Cardiometabolic risk factors**				
Systolic blood pressure, mmHg	105.2 ± 12.5^b^	104.0 ± 14.4^c^	100.4 ± 11.8^bc^	** < 0.001**
Diastolic blood pressure, mmHg	65.8 ± 11.8^b^	66.0 ± 13.6^c^	62.2 ± 11.4^bc^	** < 0.001**
Fasting plasma glucose, mg/dL	77.8 ± 8.5	77.5 ± 10.6	77.2 ± 8.9	0.759
Total cholesterol, mg/dL	163.5 [146–181]	165 [149–181]	169 [148–187]	0.280
HDL cholesterol, mg/dL	57 [49–67]	57 [49–64]	57 [49–66]	0.883
LDL cholesterol, mg/dL	94 [79–108]	96 [84–111]	94.5 [82–112.3]	0.300
Non-HDL cholesterol, mg/dL	104 [89–122]	107 [95–124]	107 [94–125.4]	0.176
Triglycerides, mg/dL	53 [44–68]	53 [43–65]	52 [43–64]	0.783

Data are expressed as mean ± SD or median [IQR] for continuous variables and percentages (numbers) for categorical variables. *p* values were calculated by the chi-square or ANOVA test for categorical and continuous variables, respectively. Values in bold indicate *p* values < 0.05, which were considered significant. Bonferroni’s test for multiple comparisons was used in those significant results calculated by analysis of covariance. Significant differences (*p* value < 0.05) between categories of adherence to the 6-healthy lifestyle behaviors composite score are expressed as follows: *a* = T1 vs.T2, *b* = T1 vs. T3, and *c* = T2 vs. T3

*BMI* body mass index, *HDL* high-density lipoprotein, *LDL* low-density lipoprotein

**Supplementary Table** **1** summarizes the dietary characteristics across tertiles of adherence to the healthy lifestyle behavior composite score. Compared to participants in the reference category (1st tertile), participants in the 3rd tertile of adherence had lower intakes of total energy, carbohydrates, sodium, other dairy products, processed, and derivatives meat products, tubers, pastries, sugar, candies, and sugary beverages (all *p* < 0.05). Children in the highest category of adherence to the composite score also reported higher intakes of protein, monounsaturated fatty acids, fiber, cheese, fish, seafood, vegetables, fruits, nuts, and whole grains (all *p* < 0.05).

The associations between the adherence to the healthy lifestyle behavior composite score and several cardiometabolic risk factors are shown in Table 3. In unadjusted models, compared to children allocated in the lowest adherence category, those in the 3rd tertile of adherence to the composite score were associated with a 60% lower risk of overweight or obesity prevalence, lower waist circumference, FMI, SBP, and DBP. In the adjusted models, these associations remained, except for DBP. In addition, an inverse association was observed between the highest tertile of adherence and fasting plasma glucose concentration [*β* coefficient (95% CI), − 1.9 (− 3.5, − 0.4); *p* = 0.013]. Interaction analyses between the composite score and sex were not statistically significant. In sensitivity analyses, the association between the composite score and FMI was not significant when children aged under 5 years were excluded, but the negative direction remained.

**Table 3:** Associations between the adherence to the composite score comprised of 6 healthy lifestyle behaviors and several cardiometabolic risk factors

	**Tertiles of adherence to the healthy lifestyle behavior composite score**		
	**T1** (< 3 points)	**T2** (3–4 points)	**T3** (> 4 points)
**Adiposity**			
*Weight status, n*^a^	392	296	250
Crude model	1 (ref.)	0.6 (0.4, 0.8) **	0.3 (0.2, 0.5) **
Adjusted model	1 (ref.)	0.6 (0.4, 0.9) *	0.4 (0.2, 0.6) **
*Waist circumference, n*^b^	388	293	247
Crude model	0 (ref.)	− 0.3 (− 1.4, 0.7)	− 1.2 (− 2.3, − 0.1) *
Adjusted model	0 (ref.)	− 0.4 (− 1.3, 0.6)	− 1.4 (− 2.5, − 0.4) **
*Fat mass index, n*^b^	342	265	222
Crude model	0 (ref.)	− 0.3 (− 0.5, − 0.1) **	− 0.5 (− 0.7, − 0.3) **
Adjusted model	0 (ref.)	− 0.2 (− 0.4, 0.1)	− 0.3 (− 0.5, − 0.1) *
**Cardiometabolic risk factors**			
*Systolic blood pressure, n*^b^	376	278	231
Crude model	0 (ref.)	− 1.2 (− 3.2, 0.8)	− 4.8 (− 6.9, − 2.7) **
Adjusted model	0 (ref.)	− 0.1 (− 2.1, 1.9)	− 3.0 (− 5.2, − 0.9) **
*Diastolic blood pressure, n*^b^	376	278	233
Crude model	0 (ref.)	0.1 (− 1.8, 2.1)	− 3.6 (− 5.6, − 1.6) **
Adjusted model	0 (ref.)	1.1 (− 0.8, 2.9)	− 2.0 (− 4.1, 0.0)
*Fasting plasma glucose, n*^b^	340	237	208
Crude model	0 (ref.)	− 0.2 (− 1.8, 1.3)	− 0.6 (− 2.2, 1.0)
Adjusted model	0 (ref.)	− 0.8 (− 2.3, 0.6)	− 1.9 (− 3.5, − 0.4) *
*Total cholesterol, n*^b^	338	237	207
Crude model	0 (ref.)	2.6 (− 2.1, 7.3)	3.3 (− 1.6, 8.1)
Adjusted model	0 (ref.)	2.3 (− 2.4, 7.0)	2.1 (− 3.0, 7.2)
*HDL cholesterol, n*^b^	336	237	207
Crude model	0 (ref.)	− 0.9 (− 3.2, 1.3)	0.3 (− 2.1, 2.6)
Adjusted model	0 (ref.)	− 1.3 (− 3.6, 0.9)	− 0.5 (− 3.0, 2.0)
*LDL cholesterol, n*^b^	314	227	196
Crude model	0 (ref.)	3.1 (− 1.1, 7.3)	1.4 (− 3.0, 5.8)
Adjusted model	0 (ref.)	2.7 (− 1.5, 7.0)	− 0.0 (− 4.5, 4.5)
*Non-HDL cholesterol, n*^b^	336	237	207
Crude model	0 (ref.)	3.9 (− 0.3, 8.1)	3.4 (− 1.0, 7.7)
Adjusted model	0 (ref.)	4.0 (− 0.2, 8.3)	3.0 (− 1.6, 7.6)
*Triglycerides, n*^b^	337	237	207
Crude model	0 (ref.)	− 1.1 (− 4.6, 2.4)	0.5 (− 3.2, 4.2)
Adjusted model	0 (ref.)	− 0.0 (− 3.5, 3.5)	2.9 (− 0.9, 6.7)

Tertiles of adherence to the composite score comprised of 6 healthy lifestyle behaviors (exposure). All models were adjusted by number of participants recruited in each recruitment center (< 130, 130–200, and > 200), sex, age and mother’s educational level (primary or lower, secondary, academic-graduate or no reported data), birth weight (low/normal/high), and maternal overweight/obesity (yes/no)

*CI* confidence interval, *HDL cholesterol*, high-density lipoprotein cholesterol, *LDL cholesterol*, low-density lipoprotein cholesterol

^*^p values < 0.05; **p values < 0.01

^a^Multivariable logistic regression model: weight status was a dichotomous outcome (underweight or normal weight (1) and overweight or obesity (2)), and results were expressed in OR (95% CI)

^b^Multivariable lineal regression models: waist circumference in cm; fat mass index in kg/m^2^; systolic and diastolic blood pressure in mmHg; and fasting plasma glucose and lipid profile in mg/dL as outcomes which results were expressed in *β* (95% CI)

**Fig. 2:**
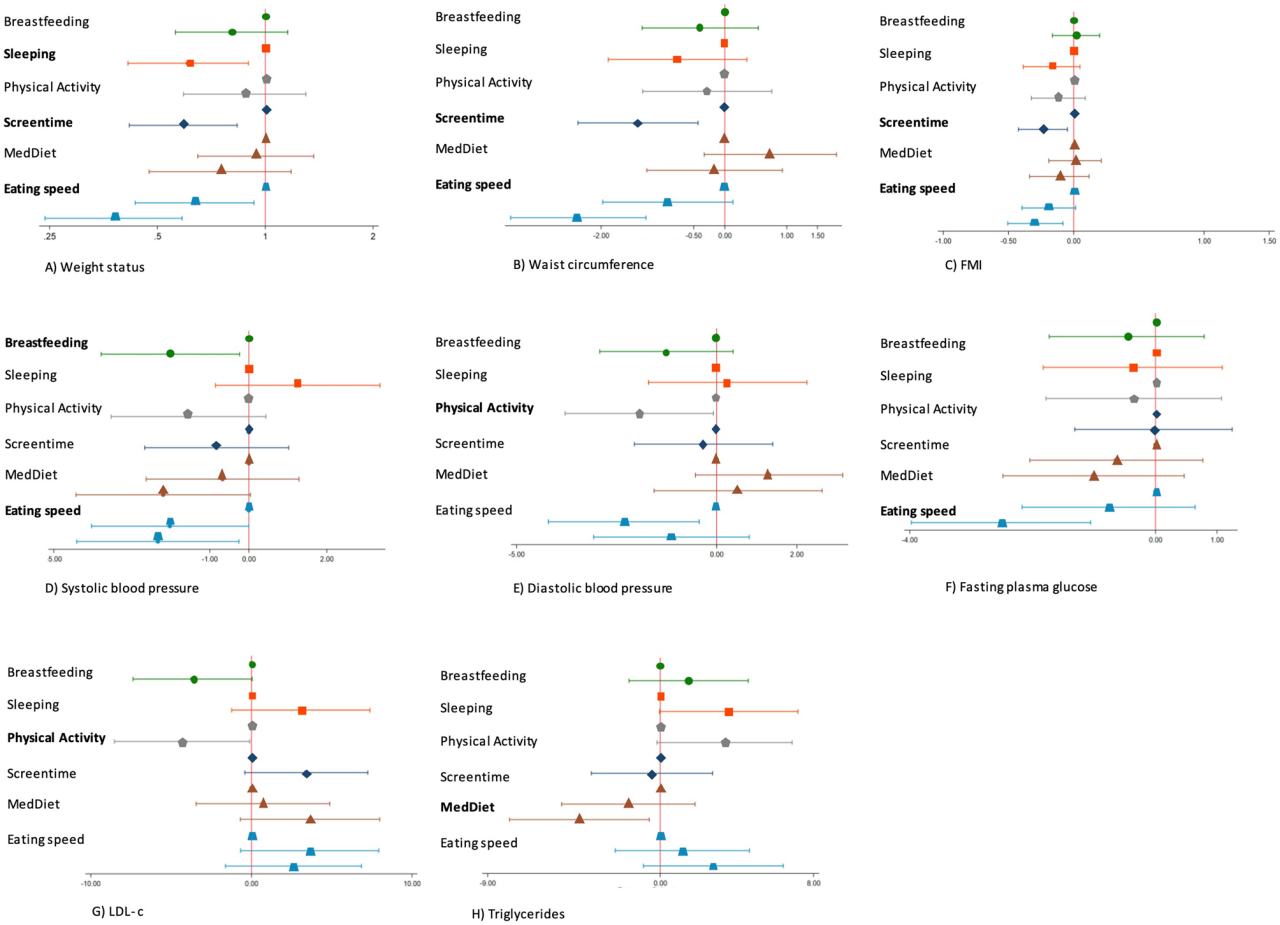
Multiple logistic regression (**A**) or linear regression models (**B–H**) and their 95% CI between compliance with each healthy lifestyle behavior (exposure) and cardiometabolic risk factors (outcomes). Models were adjusted by number of participants recruited in each recruitment center (< 130, 130–200, and > 200), sex, age and mother’s educational level (primary or lower, secondary, academic-graduate or no reported data), birth weight (low/normal/high), and maternal obesity (yes/no). Statistically significant associations are shown in bold. Breastfeeding: exclusive breastfeeding for the first 6 months (no/yes); sleeping: adequate duration for age (no/yes); physical activity: ≥ 2 h/week (no/yes); Screentime: < 2 h/day (no/yes); adherence to the Mediterranean diet: low, moderate, or high; eating speed: slow, moderate, or fast

The associations between each of the 6 healthy lifestyle behaviors and each outcome are shown in Fig. 2. An adequate sleep duration for age, screentime for < 2 h/day, and slow eating (> 85 min in main meals) were associated with a lower prevalence risk of overweight or obesity [OR (95% CI)—0.6 (0.4, 0.9); 0.6 (0.4, 0.8); and 0.4 (0.2,0.6), respectively]. Screentime for < 2 h/day and slow eating were associated with lower waist circumference [*β* (95% CI): − 1.3 (− 2.1, − 0.4) and − 2.2 (− 3.2, − 1.1), respectively] and FMI [*β* (95% CI): − 0.2 (− 0.4, − 0.0) and − 0.3 (0.5, − 0.1), respectively]. Exclusive breastfeeding for the first 6 months of age and slow eating were associated with lower SBP [*β* (95% CI): − 2.0 (− 3.8, − 0.2) and − 2.3 (− 4.4, − 0.3), respectively]. Physical activity for ≥ 2 h/week was associated with decreased levels of DBP and LDL-c [*β* (95% CI): − 1.9 (− 3.8, − 0.1) and − 4.3 (− 8.5, − 0.1), respectively]. Slow eating was associated with lower fasting plasma glucose concentration [*β* (95% CI): − 2.5 (− 4.0, − 1.1)]. Higher adherence to the MedDiet (≥ 13 points) was inversely associated with serum triglyceride levels [*β* (95% CI): − 4.2 (− 7.9, − 0.6)]. Children whose parents or caregivers reported screentime for < 2 h/day showed a higher total cholesterol and non-HDL-c (data not shown). Slow eating was associated with lower OR for overweight/obesity and *β*-coefficients for FMI and SBP, respectively. When these models were further adjusted by the other lifestyle behaviors except for the one of interest, most of the associations remained (data not shown).

## Discussion

In the present study, a higher combined adherence to the healthy behaviors of breastfeeding, sleep duration, physical activity, screentime, eating speed, and MedDiet dietary pattern was associated with lower waist circumference, FMI, SBP and fasting plasma glucose, and risk of overweight or obesity In the individual associations between each of the healthy lifestyle behaviors and the cardiometabolic risk factors assessed, slow eating speed was statistically associated to lower adiposity (weight status, waist circumference, and FMI). Moreover, eating speed was the only lifestyle behavior associated with lower fasting plasma glucose concentration. Additionally, participants in the highest tertile of adherence to the healthy lifestyle behavior composite score showed a lower energy intake and a dietary pattern closer to the MedDiet, and their mothers had a higher educational level, lower BMI, and overweight/obesity status.

Some observational studies [12–14] but not all [13, 15] are in line with our results, as they reported associations between higher adherence to a healthy lifestyle pattern and lower adiposity in children of similar age [13]. In contrast with our results, no significant associations were reported for blood pressure, HDL-c or serum triglyceride levels, and fasting plasma glucose concentration was not assessed. Discrepancies between studies could be partially explained by differences in the lifestyle behaviors assessment. Bawaked et al. [13] conducted cross-sectional and longitudinal analyses in Spanish children in which a 5-item score was created. They categorized the score according to tertiles and assigned a different punctuation to each tertile, based on whether the behavior was favorable (extracurricular physical activity, sleep duration, and plant-based food consumption) or unfavorable (screentime and consumption of ultra‐processed food) [13]. It should be considered that lifestyle behavior recommendations might differ according to age (e.g., sleep duration) and using an alternative method could lead to discrepancy. Furthermore, the high prevalence of children with overweight or obesity may bias the assessment of lifestyle behaviors. In addition, the aforementioned studies [12–15] have been conducted in high-income countries, and therefore, the results may differ substantially from other studies conducted in low or middle-income countries. Moreover, previous studies have not assessed a complete dietary pattern [12–14], eating speed, adherence to the MedDiet, or breastfeeding [12–15].

Similarly, studies in European children [13, 14, 27] have also reported negative associations between physical activity and adiposity. In contrast, lower triglyceride levels were observed in Greek children exercising for > 2 h/week [28]. Nevertheless, compliance with physical activity recommendations is scarcely assessed [14, 27].

The evidence on associations between sleep duration and adiposity or cardiometabolic disorders in children is inconsistent; yet certain observational studies [13, 27] are in line with our results. However, no significant associations were reported for adiposity in children from 8 European countries [14], and sleep duration was inversely associated with SBP in the long-term [13]. Potential effects from sleep duration on energy expenditure [29] and the nervous system [30] have been suggested, which may regulate adiposity and blood pressure, respectively.

Previous observational studies conducted in preschool [13, 14] and school children [14] also observed positive associations between screentime and adiposity. Nevertheless, a meta-analysis [31] reported that there is insufficient evidence on this relationship. However, longer screentime could limit the time spent on physical activities [32] and explain adiposity outcomes. Regarding the unexpected results we observed for the screentime, a cross-sectional study [33] in Australian school children reported no significant associations between sedentary behaviors and lipids; however, recommendations were not assessed.

Evidence on eating speed and cardiometabolic risk in children is scarce [34–37]. However, similar associations between slow eating and adiposity were reported in a Finnish cross-sectional study [34] and an American clinical trial [35] but other cardiometabolic risk factors were no assessed. In this sense, we cannot discard a role by diet quality since it has been suggested that dietary energy density and eating speed could regulate energy intake [38].

Previous studies have demonstrated the benefits of the MedDiet on cardiometabolic profile in adults [39–42] but not in children [28, 43]. In the present study, similar to evidence in adults [35, 38], MedDiet was inversely related to serum triglyceride levels, which could be explained by some MedDiet characteristics [39], rich in fish, nuts, olive-oil, legumes, and other plant-based foods but low in refined cereals and sugar [44].

Regarding exclusive breastfeeding, a meta-analysis [45] also reported negative association with childhood obesity. However, a clinical trial [46] conducted in Belarussian infants reported no effect on decreasing blood pressure or risk of obesity in adolescence. Regarding this relationship, it exists insufficient solid evidence [47], and therefore, further studies are warranted.

The healthy lifestyle behavior composite score has been developed based on current evidence. However, possible interactions among the lifestyle behaviors assessed in the present study cannot be disregarded. For example, short sleep duration and high screentime or noncompliance with physical activity recommendations have been observed in association with higher adiposity in children [27]. Additionally, an interaction between diet quality and physical activity was also reported, for which an unhealthy diet showed significant differences according to the level of physical activity [27]. Furthermore, additional putative lifestyle behaviors might have coexisted in participants that partially explain the results observed.

The present study has some limitations that deserve to be mentioned: (a) this is a cross-sectional study, so cause-effect conclusions should not be made; (b) the studied population corresponded to Spanish preschool children so results cannot be extrapolated to other populations; (c) residual confounding or undetected cardiometabolic disorders due to early age in the studied population cannot be dismissed; (d) equations for bioelectrical bioimpedance have not been validated in children under 5 years of age so, sensitivity analyses were performed; and (e) moderate-vigorous physical activities were not identified by the questionnaires, and therefore, we have used the WHO cutoff point [22]*.* The present study also has strengths that must be highlighted. First, a large sample size from 7 Spanish centers, drawn from the general population, was studied. Second, data from blood samples collected were available for the final studied population. Third, several measures were considered to assess adiposity and cardiometabolic risk, as well as multiple confounders.

## Conclusions

Higher adherence to the healthy behavior composite score and therefore to 6 healthy lifestyle behaviors was associated with lower cardiometabolic risk in a population of preschool children. Slow eating speed was individually related to most of the cardiometabolic risk factors. If further research could confirm these associations, this composite score could become a useful clinical tool that may contribute to enhance the prevention of adiposity and cardiometabolic disorders by detecting detrimental lifestyle behaviors since early childhood.

### Supplementary Information

Below is the link to the electronic supplementary material.

Supplementary file1 (DOCX 41.8 KB)

Supplementary file2 (DOCX 23.9 KB)

## Data Availability

The datasets generated and analyzed during the current study are not publicly available due to data regulations and for ethical reasons, considering that this information might compromise research participants’ consent because our participants only gave their consent for the use of their data by the original team of investigators. However, collaboration for data analyses can be requested by sending a letter to the CORALS steering Committee (estudiocoral@corals.es). The request will then be passed to all the members of the CORALS Steering Committee for deliberation.

## Abbreviations

CORALS: Childhood Obesity Risk Assessment Longitudinal Study
*β*: Beta coefficient
CI: Confidence interval
WHO: World Health Organization
ALADINO: ALimentación, Actividad Física, Desarrollo INfantil y Obesidad
MedDiet: Mediterranean diet
FMI: Fat mass index
HDL-c: High-density lipoprotein cholesterol
LDL-c: Low-density lipoprotein cholesterol
FFQ: Food frequency questionnaire
IQR: Interquartile ratio
OR: Odds ratio
BMI: Body mass index
ISCIII: Instituto Salud Carlos III
SBP: Systolic blood pressure
DBP: Diastolic blood pressure
