# Supplementary material for: Adherence to a healthy lifestyle behavior composite score and cardiometabolic risk factors in Spanish children from the CORALS cohort
Source: Eur J Pediatr. 2024 Jan 23;183(4):1819–30. doi: 10.1007/s00431-023-05389-z (PMC11001667; doi:10.1007/s00431-023-05389-z)
Supplement: Supplementary file 1 — Supplementary file1 (DOCX 41.8 KB) [file 431_2023_5389_MOESM1_ESM.docx]

**ADHERENCE TO A HEALTHY LIFESTYLE BEHAVIOR COMPOSITE SCORE AND CARDIOMETABOLIC RISK FACTORS IN SPANISH CHILDREN FROM THE CORALS COHORT**

Tany E. Garcidueñas-Fimbres^1,2,3^, MD, PhD; Carlos Gómez-Martínez^1,2,3^, MSc; Maria Pascual-Compte^1,2^, MSc; Jose Manuel Jurado-Castro^3,4,^, PhD; Rosaura Leis^3,5,6,^ MD, PhD; Luis A. Moreno^3,7,8^, MD, PhD; Santiago Navas-Carretero^3,9,10^, PhD; Pilar Codoñer-Franch^3,11^, MD, PhD; Ana Moreira Echeverria^12^, MD; Belén Pastor-Villaescusa^4^, PhD; Alicia López-Rubio^5,6^, MSc; Sara Moroño García^13^, RN; Pilar De Miguel-Etayo^3,7,8^, PhD; J. Alfredo Martínez^3,9^, MD, PhD; Inmaculada Velasco Aguayo^4^, PhD; Rocío Vázquez-Cobela^3,5,6^, PhD; Joaquín Escribano^2,14^, MD, PhD; María Luisa Miguel-Berges^3,7,8^, PhD; María José De La Torre-Aguilar^4^, MD, PhD; Mercedes Gil-Campos^3,4^, MD, PhD; Jordi Salas-Salvadó^1,2,3*^, MD, PhD; Nancy Babio^1,2,3*^.

**Affiliations**

^1^Universitat Rovira i Virgili, Departament de Bioquímica i Biotecnologia, Unitat de Nutrició Humana. Reus, Spain

^2^Institut d'Investigació Sanitària Pere Virgili (IISPV) Reus, Spain.

^3^Centro de Investigación Biomédica en Red de Fisiopatología de la Obesidad y Nutrición (CIBEROBN), Instituto de Salud Carlos III (ISCIII), Madrid, Spain.

^4^Metabolism and Investigation Unit, Reina Sofia University Hospital. Maimonides Biomedical Research Institute of Cordoba (IMIBIC). University of Córdoba; Córdoba 14004, Spain.

^5^Unit of Pediatric Gastroenterology, Hepatology and Nutrition. Pediatric Service. Hospital Clínico Universitario de Santiago. 15706. Santiago de Compostela, Spain.

^6^Pediatric Nutrition Research Group, Health Research Institute of Santiago de Compostela (IDIS). Unit of Investigation in Nutrition, Growth and Human Development of Galicia-USC. 15706 Santiago de Compostela, Spain.

^7^Growth, Exercise, Nutrition and Development (GENUD) Research Group, University of Zaragoza, Zaragoza, Spain.

^8^Instituto Agroalimentario de Aragón (IA2). Instituto de Investigación Sanitaria de Aragón (IIS Aragón), Zaragoza, Spain.

^9^University of Navarra, Center for Nutrition Research, 31008 Pamplona, Spain; University of Navarra, Fac Pharm & Nutr, Dept Nutr Food Sci & Physiol, 31008, Pamplona, Spain.

^10^IdisNA, Navarra Institute for Health Research, Pamplona, Spain.

^11^Dr. Peset University Hospital. Department of Pediatrics, Obstetrics and Gynecology, University of Valencia, Valencia, Spain.

^12^Fundació Hospital Sant Joan de Deu Martorell, Barcelona, Spain.

^13^EDP Salut Sant Joan Baix Camp-ABS Riudoms, Riudoms, Spain.

^14^Paediatrics, Nutrition, and Development Research Unit, Hospital Universitari Sant Joan de Reus. Universitat Rovira i Virgili, Reus, Spain.

*Corresponding authors: **Nancy Babio** [nancy.babio@urv.cat] and **Jordi Salas-Salvadó** [jordi.salas@urv.cat], Unitat de Nutrició Humana, Departament de Bioquímica i Biotecnología, Universitat Rovira i Virgili. Tel: +34 977 75 93 13, Fax: +34 977 75 93 22.

**SUPPLEMENTARY MATERIAL**

**APPENDIX 1 – Additional study assessments**

For the purposes of this study, additional variables were assessed including:

*Early life factors.*

Weight gain during pregnancy (kg) and birth weight (kg) were assessed. Birth weight was categorized in low (<2.5 kg), normal (2.5-3.9 kg) and high (>4 kg) [1].

*Maternal factors.*

The level of education (primary or lower, secondary or academic-graduated or no reported), socio-professional category (homemaker/student/retired/unemployed or employee) and self-reported anthropometric measures were considered. The maternal BMI (kg/m^2^) was estimated and categorized [2].

*Diet.*

A trained dietitian completed a semi-quantitative 125-item FFQ for each participant. Total energy and the intake of nutrients were estimated according to Spanish nutritional composition databases [3–5]. Implausible energy intake was assessed according to Goldberg cut-offs adapted to children [6] and those participants with missing data or implausible reported energy intake were no included in final analyses.

**APPENDIX 2 – Additional statistical analyses**

CORALS database updated to December 2021 was used for the present cross-sectional study. One-factor ANOVA, Kruskal Wallis and chi-square tests were used to describe differences in baseline characteristics according to tertiles of adherence to the healthy lifestyle behavior composite score, which were reported as mean ± SD or median [interquartile range (IQR)] for continuous variables and as percentage (numbers) for categorical variables. Bonferroni’s tests for multiple comparison were used in those statistically significant one-factor ANOVA analyses. Missing data confounders <10% on covariates data was imputed to the mean [7]. Interaction analyses were conducted by sex. Due to the lack of validation of bioimpedance equations in children under 5-year-old, a FMI sensitivity analysis was conducted excluding these children.

**References**

1. Yerushalmy J (1967) The classification of newborn infants by birth weight and gestational age. J Pediatr 71:164–172

2. World Health Organ Tech Rep Ser. (2000) Obesity: Preventing and Managing the Global Epidemic: report of a WHO consultation.

3. Farran A, Zamora R, Cervera P, Centre d’Enseyament Superior de Nutrició i Dietètica (2004) Tablas de composición de alimentos del CESNID, 3rd ed.

4. Babio Sánchez N, de la Serra Besora Moreno M, Lladó Bellette N, Salas Salvadó Jordi (2018) CELIACBASE. Tabla de composición de productos sin gluten. Publicacions URV

5. Babio Nancy, Dragusan Lica Natalia, De Las Heras Sara, Blanquer Maria (2022) Tabla de Composición de Productos y Alternativas Vegetales, 1st ed. Publicacions URV, Reus

6. Börnhorst C, Huybrechts I, Ahrens W, et al (2013) Prevalence and determinants of misreporting among European children in proxy-reported 24 h dietary recalls. British Journal of Nutrition 109:1257–1265

7. Ton de Waal, Jeroen Pannekoek, Sander Scholtus (2011) Statistical Data Editing and Imputation.
