# Supplementary material for: Adherence to a healthy lifestyle behavior composite score and cardiometabolic risk factors in Spanish children from the CORALS cohort
Source: Eur J Pediatr. 2024 Jan 23;183(4):1819–30. doi: 10.1007/s00431-023-05389-z (PMC11001667; doi:10.1007/s00431-023-05389-z)
Supplement: Supplementary file 2 — Supplementary file2 (DOCX 23.9 KB) [file 431_2023_5389_MOESM2_ESM.docx]

**ADHERENCE TO A HEALTHY LIFESTYLE BEHAVIOR COMPOSITE SCORE AND CARDIOMETABOLIC RISK FACTORS IN SPANISH CHILDREN FROM THE CORALS COHORT**

Tany E. Garcidueñas-Fimbres^1,2,3^, MD, PhD; Carlos Gómez-Martínez^1,2,3^, MSc; Maria Pascual-Compte^1,2^, MSc; Jose Manuel Jurado-Castro^3,4,^, PhD; Rosaura Leis^3,5,6,^ MD, PhD; Luis A. Moreno^3,7,8^, MD, PhD; Santiago Navas-Carretero^3,9,10^, PhD; Pilar Codoñer-Franch^3,11^, MD, PhD; Ana Moreira Echeverria^12^, MD; Belén Pastor-Villaescusa^4^, PhD; Alicia López-Rubio^5,6^, MSc; Sara Moroño García^13^, RN; Pilar De Miguel-Etayo^3,7,8^, PhD; J. Alfredo Martínez^3,9^, MD, PhD; Inmaculada Velasco Aguayo^4^, PhD; Rocío Vázquez-Cobela^3,5,6^, PhD; Joaquín Escribano^2,14^, MD, PhD; María Luisa Miguel-Berges^3,7,8^, PhD; María José De La Torre-Aguilar^4^, MD, PhD; Mercedes Gil-Campos^3,4^, MD, PhD; Jordi Salas-Salvadó^1,2,3*^, MD, PhD; Nancy Babio^1,2,3*^.

**Affiliations**

^1^Universitat Rovira i Virgili, Departament de Bioquímica i Biotecnologia, Unitat de Nutrició Humana. Reus, Spain

^2^Institut d'Investigació Sanitària Pere Virgili (IISPV) Reus, Spain.

^3^Centro de Investigación Biomédica en Red de Fisiopatología de la Obesidad y Nutrición (CIBEROBN), Instituto de Salud Carlos III (ISCIII), Madrid, Spain.

^4^Metabolism and Investigation Unit, Reina Sofia University Hospital. Maimonides Biomedical Research Institute of Cordoba (IMIBIC). University of Córdoba; Córdoba 14004, Spain.

^5^Unit of Pediatric Gastroenterology, Hepatology and Nutrition. Pediatric Service. Hospital Clínico Universitario de Santiago. 15706. Santiago de Compostela, Spain.

^6^Pediatric Nutrition Research Group, Health Research Institute of Santiago de Compostela (IDIS). Unit of Investigation in Nutrition, Growth and Human Development of Galicia-USC. 15706 Santiago de Compostela, Spain.

^7^Growth, Exercise, Nutrition and Development (GENUD) Research Group, University of Zaragoza, Zaragoza, Spain.

^8^Instituto Agroalimentario de Aragón (IA2). Instituto de Investigación Sanitaria de Aragón (IIS Aragón), Zaragoza, Spain.

^9^University of Navarra, Center for Nutrition Research, 31008 Pamplona, Spain; University of Navarra, Fac Pharm & Nutr, Dept Nutr Food Sci & Physiol, 31008, Pamplona, Spain.

^10^IdisNA, Navarra Institute for Health Research, Pamplona, Spain.

^11^Dr. Peset University Hospital. Department of Pediatrics, Obstetrics and Gynecology, University of Valencia, Valencia, Spain.

^12^Fundació Hospital Sant Joan de Deu Martorell, Barcelona, Spain.

^13^EDP Salut Sant Joan Baix Camp-ABS Riudoms, Riudoms, Spain.

^14^Paediatrics, Nutrition, and Development Research Unit, Hospital Universitari Sant Joan de Reus. Universitat Rovira i Virgili, Reus, Spain.

*Corresponding authors: **Nancy Babio** [nancy.babio@urv.cat] and **Jordi Salas-Salvadó** [jordi.salas@urv.cat], Unitat de Nutrició Humana, Departament de Bioquímica i Biotecnología, Universitat Rovira i Virgili. Tel: +34 977 75 93 13, Fax: +34 977 75 93 22.

| **Supplemental Table 1.** Dietary characteristics of the study participants across categories of adherence to the composite score comprised of 6 healthy lifestyle behaviors. | | | | |
| --- | --- | --- | --- | --- |
|  | **Tertiles of adherence to the healthy lifestyle behavior composite score** | | | |
|  | **T1** (<3 points)  n=392 | **T2** (3-4 points)  n=296 | **T3** (>4 points)  n=250 | **p value** |
| **Dietary intake contribution** |  |  |  |  |
| Total energy intake, kcal/day | 1752 ± 378^ab^ | 1674 ± 352^a^ | 1642 ± 304^b^ | **<0.001** |
| Carbohydrates, % of total energy intake | 43.5 ± 5.7^b^ | 42.7 ± 5.0 | 42.2 ± 5.6^b^ | **0.009** |
| Proteins, % of total energy intake | 14.7 ± 2.1^ab^ | 15.2 ± 2.1^a^ | 15.2 ± 2.1^b^ | **<0.001** |
| Protein intake per body weight, g/kg/day | 3.2 ± 1.0 | 3.3 ± 0.9 | 3.4 ± 0.9 | 0.058 |
| Total fat, % of total energy intake | 41.9 ± 5.8 | 42.1 ± 5.4 | 42.6 ± 6.3 | 0.293 |
| Saturated fatty acids, % of total energy intake | 14.0 ± 2.0 | 14.0 ± 2.0 | 13.8 ± 2.0 | 0.579 |
| Monounsaturated fatty acids, % of total energy intake | 18.5 ± 4.4^b^ | 19.0 ± 4.1 | 19.6 ± 5.0^b^ | **0.013** |
| Polyunsaturated fatty acids, % of total energy intake | 6.3 ± 1.9 | 6.0 ± 1.4 | 6.0 ± 1.5 | **0.034** |
| Fiber, g/1,000 kcal | 8.0 ± 2.1^ab^ | 8.5 ± 2.3^ac^ | 9.2 ± 2.5^bc^ | **<0.001** |
| ≥14 gr/1000 kcal, % (n) | 1.3 (5) | 3.4 (10) | 6.4 (16) | **0.002** |
| Sodium, mg/dL | 2383 ± 737^b^ | 2322 ± 694 | 2182 ± 672^b^ | **0.002** |
| **Dairy products** |  |  |  |  |
| Milk, g/day | 329.4 ± 227.8 | 331.1 ± 242.9 | 291.8 ± 216.1 | 0.078 |
| Yogurt, g/day | 110.4 ± 98.4 | 118.1 ± 97.2 | 104.1 ± 80.1 | 0.214 |
| Cheese, g/day | 11.9 ± 12.0^b^ | 11.5 ± 12.6^c^ | 14.6 ± 11.3^bc^ | **0.006** |
| Other dairy products, g/day | 104.3 ± 102.9^ab^ | 75.7 ± 85.4^a^ | 64.9 ± 73.1^b^ | **<0.001** |
| **Protein foods** |  |  |  |  |
| White meat, g/day | 24.2 ± 9.5 | 23.9 ± 9.8 | 24.7 ± 8.2 | 0.598 |
| Unprocessed red meat, g/day | 18.8 ± 13.6 | 19.3 ± 11.9 | 18.6 ± 12.6 | 0.809 |
| Processed and derivatives meat products, g/day | 26.6 ± 16.2^b^ | 25.2 ± 14.6 | 23.5 ± 13.0^b^ | **0.036** |
| Egg, g/day | 22.7 ± 9.8 | 24.4 ± 9.1 | 23.3 ± 8.3 | 0.053 |
| Fish and seafood, g/day | 30.6 ± 17.7^ab^ | 35.8 ± 17.3^a^ | 36.3 ± 17.0^b^ | **<0.001** |
| **Vegetables and fruits** |  |  |  |  |
| Vegetables, g/day | 70.6 ± 53.6^b^ | 75.3 ± 47.3^c^ | 88.3 ± 51.6^bc^ | **<0.001** |
| Tubers, g/day | 42.8 ± 20.0^b^ | 40.0 ± 19.7 | 37.6 ± 17.9^b^ | **0.003** |
| Fruits, g/day | 163.2 ± 114.1^ab^ | 193.2 ± 113.1^ac^ | 217.9 ± 130.4^bc^ | **<0.001** |
| **Nuts** |  |  |  |  |
| Nuts, g/day | 3.1 ± 4.2^b^ | 3.6 ± 5.3 | 4.5 ± 5.4^b^ | **0.001** |
| **Cereals and Legumes** |  |  |  |  |
| Legumes, g/day | 14.5 ± 8.3 | 13.6 ± 6.1 | 14.1 ± 6.1 | 0.248 |
| Refined cereals, g/day | 74.5 ± 36.9 | 70.8 ± 32.4 | 71.1 ± 36.2 | 0.312 |
| Whole cereals, g/day | 5.8 ± 13.2^b^ | 8.2 ± 15.0^c^ | 11.4 ± 18.8^bc^ | **<0.001** |
| **Miscellaneous** |  |  |  |  |
| Oil and fats, g/day | 26.0 ± 15.9 | 25.9 ± 14.6 | 27.8 ± 16.8 | 0.303 |
| Pastries, g/day | 43.5 ± 31.8^b^ | 39.0 ± 28.6 | 35.9 ± 25.1^b^ | **0.005** |
| Sugars and candies, g/day | 14.9 ± 11.4^ab^ | 12.8 ± 10.6^a^ | 11.1 ± 9.1^b^ | **<0.001** |
| **Beverages** |  |  |  |  |
| Water, ml/day | 855.3 ± 359.4 | 846.2 ± 359.8 | 865.4 ± 409.9 | 0.837 |
| Sugary beverages, ml/day | 132.3 ± 145.8^ab^ | 101.1 ± 109.2^a^ | 92.5 ± 107.9^b^ | **<0.001** |
| Tea and infusions, ml/day | 6.3 ± 27.6 | 7.6 ± 33.4 | 6.9 ± 26.1 | 0.855 |
| Data are expressed as mean ± SD.  P-values were calculated by the ANOVA. The p values <0.05 were considered significant.  The Bonferroni’s test for multiple comparisons was used for those significant results calculated by analysis of covariance. Significant differences (p value <0.05) between categories of adherence to the 6-healthy lifestyle behaviors composite score are expressed as: a=T1vs. T2; b=T1 vs. T3 and c=T2 vs. T3. | | | | |
